# Dual Thermo- and pH-Responsive Polymer Nanoparticle Assemblies for Potential Stimuli-Controlled Drug Delivery

**DOI:** 10.1021/acsabm.4c01167

**Published:** 2024-12-11

**Authors:** Sára Pytlíková, Rafal Konefał, Robert Pola, Alena Braunová, Volodymyr Lobaz, Miroslav Šlouf, Hynek Beneš, Daniil Starenko, Kateřina Běhalová, Marek Kovář, Tomáš Etrych, Richard Laga, Michal Pechar

**Affiliations:** † Institute of Macromolecular Chemistry, Czech Academy of Sciences, Heyrovského nám. 2, Prague 6, 162 00, Czech Republic; ‡ Institute of Microbiology, Czech Academy of Sciences, Vídeňská 1083, Prague 4, 142 00, Czech Republic

**Keywords:** thermoresponsive polymers, pH-sensitive polymers, self-assembling block copolymers, drug delivery systems, RAFT polymerization

## Abstract

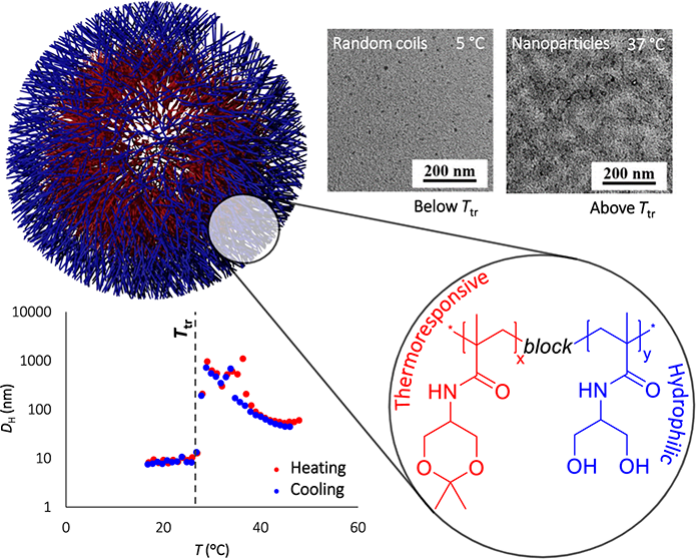

The development of stimuli-responsive drug delivery systems enables targeted delivery and environment-controlled drug release, thereby minimizing off-target effects and systemic toxicity. We prepared and studied tailor-made dual-responsive systems (thermo- and pH-) based on synthetic diblock copolymers consisting of a fully hydrophilic block of poly[*N*-(1,3-dihydroxypropyl)methacrylamide] (poly(DHPMA)) and a thermoresponsive block of poly[*N*-(2,2-dimethyl-1,3-dioxan-5-yl)methacrylamide] (poly(DHPMA-acetal)) as drug delivery and smart stimuli-responsive materials. The copolymers were designed for eventual medical application to be fully soluble in aqueous solutions at 25 °C. However, they form well-defined nanoparticles with hydrodynamic diameters of 50–800 nm when heated above the transition temperature of 27–31 °C. This temperature range is carefully tailored to align with the human body’s physiological conditions. The formation of the nanoparticles and their subsequent decomposition was studied using dynamic light scattering (DLS), transmission electron microscopy (TEM), isothermal titration calorimetry (ITC), and nuclear magnetic resonance (NMR). ^1^H NMR studies confirmed that after approximately 20 h of incubation at pH 5, which closely mimics tumor microenvironment, approximately 40% of the acetal groups were hydrolyzed, and the thermoresponsive behavior of the copolymers was lost. This smart polymer response led to disintegration of the supramolecular structures, possibly releasing the therapeutic cargo. By tuning the transition temperature to the values relevant for medical applications, we ensure precise and effective drug release. In addition, our systems did not exhibit any cytotoxicity against any of the three cell lines. Our findings underscore the immense potential of these nanoparticles as eventual advanced drug delivery systems, especially for cancer therapy.

## 1 Introduction

Supramolecular polymeric self-assemblies, including micelles, polymerosomes, polyplexes, etc., are frequently studied in the field of drug delivery and targeted drug release, especially in anticancer therapy.^1−3^ Thanks to their increased hydrodynamic size, typically in the range from ten to two hundred nanometers, they are preferentially accumulated in solid tumors due to the enhanced permeability and retention (EPR) effect.^4,5^ In addition, they can provide protection to the encapsulated drugs during transport and improve the solubility of hydrophobic drugs. Their noncovalent character guarantees the gradual elimination of the polymer material from the organism due to the equilibrium between the unimer and the supramolecular structure. The internal structure of drug delivery systems can be designed to respond to various physicochemical stimuli, such as temperature,^6^ pH,^7−10^ reactive oxygen species,^11^ or light of the right wavelength.^12,13^ This response can be a prerequisite for nanoparticle formation or it can enhance its disintegration, leading to faster drug release in a specific microenvironment.

The preparation of traditional polymeric micelles or polymerosomes consisting of hydrophobic and hydrophilic polymer blocks that are irresponsive to stimuli usually requires some time-consuming procedures like evaporation of organic cosolvents from aqueous solutions under reduced pressure, dialysis from organic solvents against water, ultrasonication, etc.^14^ The use of thermoresponsive copolymers avoids these complicated techniques, because they self-assemble into supramolecular structures simply by heating their solution above the phase separation temperature.^15,16^ Moreover, the presence of suitable environmentally sensitive (e.g., hydrolytically, enzymatically, reductively, etc.) groups in the polymer structure can be exploited to ensure the gradual disassembly of the supramolecular structures, enabling excretion of the polymer from the organism.^17^

Various block copolymers have been used for the preparation of polymeric micelles and nanoparticles for drug delivery applications. Block copolymers consisting of poly(ethylene glycol) (PEG) and poly(amino acids), such as PEG-*b*-poly(β-benzyl-l-aspartate)^18^ or PEG-*b*-poly(γ-benzyl-l-glutamate),^19^ belong to the most studied types of copolymers. Similarly, PEG-polyester amphiphilic copolymers are also important micelle-forming copolymers. These include, e.g., PEG-*b*-poly(lactic acid)^20^ and PEG-*b*-poly(ε-caprolactone).^21^ Polyethers, such as poly(ethylene glycol)-*b*-poly(propylene glycol),^22^ represent another class of polymers that can be used for the preparation of nanoparticles for drug delivery. Recently, more sophisticated stimuli-sensitive block copolymers have been described, e.g., poly(*N*-isopropylacrylamide)-*block*-poly(*N*,*N*-diethylaminoethylacrylamide),^23^ poly(2-ethyl-2-oxazoline)-*block*-poly(l-lactide),^24^ poly(*N*,*N*-dimethylacrylamide)-*block*-poly(acrylamide-*co*-acrylonitrile),^6^ and poly(2-(dimethylamino)ethyl methacrylate-*co*-2-hydroxypropyl methacrylate)-*block*-poly(oligoethylene glycolmethacrylate),^25^ respectively, prepared by polymerization-induced self-assembly (PISA) in water.

Block copolymers exhibiting both thermoresponsive and pH-responsive behaviors were also reported. These systems were mostly based on acrylates consisting of a thermoresponsive and acid- sensitive block of (2,2-dimethyl-1,3-dioxolane-4-yl) acrylate (DMDA) and a hydrophilic block, e.g., 2-hydroxyethyl acrylate (HEA)^26^ or methoxy tri(ethylene glycol) acrylate (mTEGA).^27,28^ These copolymers were prepared via controlled radical polymerization (RAFT) and had relatively low dispersity. Nevertheless, the ester bonds in the structures might undergo unwanted hydrolysis in aqueous solutions. Moreover, liquid monomers, such as HEA, are very difficult to purify, especially from eventual diacryloylated cross-linkers, which lead to polymers with a broader molecular weight distribution. In our work, we focus on more hydrolytically stable amide monomers that are crystallizable.

It has been repeatedly reported^29,30^ that the ratio between the length of the hydrophilic and thermoresponsive blocks in the copolymer may substantially influence the morphology of the resulting supramolecular assemblies. Generally, the copolymers with longer hydrophilic blocks tend to form polymeric micelles, while copolymers with longer hydrophobic (or thermoresponsive) blocks prefer to self-assemble into vesicles or polymerosomes. Therefore, in this study, we compared diblock copolymers differing in the ratio between the hydrophilic and the thermoresponsive blocks.

In this work, diblock copolymers consisting of a fully hydrophilic block of poly[*N*-(1,3-dihydroxypropyl)methacrylamide] (poly(DHPMA)) and a thermoresponsive block of poly[*N*-(2,2-dimethyl-1,3-dioxan-5-yl)methacrylamide] (poly(DHPMA-acetal)) were prepared. The copolymers were synthesized using controlled radical polymerization (RAFT technique) with the intention to prepare micelles or nanoparticles with both thermoresponsive and pH-sensitive behavior.

To study temperature-induced phase separation of the prepared polymers in aqueous solutions, we used ^1^H NMR spectroscopy, ^1^H spin–spin relaxation time measurement (temperature and time dependences), and 2D nuclear Overhauser effect spectroscopy (NOESY) at various temperatures (applied only to the block copolymer) in combination with dynamic light scattering (DLS), isothermal titration calorimetry (ITC), and transmission electron microscopy (TEM).

We believe that these thermoresponsive and pH-responsive micelles and nanoparticles can be potentially utilized as sophisticated systems suitable for the delivery of various drugs, including cancerostatics. The hydrolysis of the acetal groups, which results in loss of thermoresponsive behavior and subsequent disintegration of the nanoparticles, would guarantee not only site-specific release of the drug (e.g., in a tumor tissue) from the carrier but also the excretion of the polymers in the form of unimers from the organism via glomerular filtration.

## 2 Materials and Methods

### 2.1 Chemicals

Methacryloyl chloride, 2-amino-1,3-propanediol, 2,2-dimethoxypropane, poly(styrene)-bound *p*-toluenesulfonic acid, tetrahydrofuran (THF), hexane, *N,N*-dimethylformamide (DMF), and dimethyl sulfoxide (DMSO) were purchased from Merck (Prague, Czech Republic). 2,2′-Azobis(4-methoxy-2,4-dimethylvaleronitrile) (V-70) was purchased from Wako Chemicals Europe (Neuss, Germany). Methacryloyl chloride was distilled under reduced pressure. THF was dried with calcium hydride and distilled. DMSO was dried using molecular sieves. All aqueous solutions were prepared with water purified by reverse osmosis. All other chemicals were of analytical grade. Human blood plasma, a mixture from five donors stabilized with citrate, was provided by the Institute of Haematology and Blood Transfusion (Prague, Czech Republic).

### 2.2 Size-Exclusion Chromatography (SEC)

Determination of the number- and weight-average molecular weights (*M*_n_ and *M*_w_) and dispersity (*Đ*) of the homopolymers and diblock copolymers was performed using an HPLC system (Shimadzu, Kyoto, Japan) on a TSKgel G3000 SW_XL_ column (Tosoh Bioscience, Tokyo, Japan) in a mixture of 80% methanol/20% acetate buffer (0.15 M, pH 6.5) equipped with an external multiangle light scattering (MALS) detector DAWN Helios-II and differential refractometric (dRI) detector Optilab (all from Wyatt Technology Corp., Goleta, CA, USA) at a flow rate of 0.5 mL min^–1^. The data were analyzed using the ASTRA VI software, and the refractive index increment values (d*n/*d*c*) of 0.167 mL g^–1^ for DHPMA-acetal-based homopolymers and of 0.160 mL g^–1^ for diblock copolymers, calculated as an average of values for poly(DHPMA-acetal) and poly(DHPMA), were applied for the calculation of *M*_n_, *M*_w_, and *Đ*.

### 2.3 Synthesis of Monomers and Chain Transfer Agent (CTA)

Syntheses of the monomer *N*-(1,3-dihydroxypropyl)methacrylamide (DHPMA)^31^ and a chain transfer agent (CTA) *S*-2-cyano-2-propyl-*S′*-ethyl trithiocarbonate (TTC-AIBN)^32^ were performed as described previously.

**Scheme 1:**
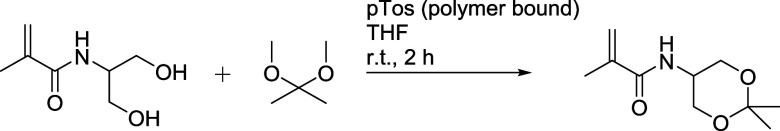
Synthesis of DHPMA-Acetal Monomer

The DHPMA-acetal (Scheme 1) was prepared by the similar procedure as reported by Huang^16^ with slight modification. DHPMA (250 mg, 1.57 mmol), 2,2-dimethoxypropane (2.2 mL, 0.018 mol), and poly(styrene)-bound *p*-toluenesulfonic acid (50 mg, 0.125 mmol) were dissolved in dry THF (4.4 mL) in the presence of inhibitor of polymerization (1,1,3,3-tetramethylbutyl)pyrocatechol). The reaction solution was stirred at 25 °C for 2 h. After filtration and removal of the solvent on a rotary evaporator under reduced pressure, the product was crystallized from hexane/THF (2:1) to afford white crystals (217 mg, 1.10 mmol, yield: 69%, mp: 105–108 °C (lit.^1^: 108 °C)), C: 59.6% (theor. 60.3%) H: 8.7% (theor. 8.6%) N: 7.2% (theor. 7.0%). ^1^H NMR 400 MHz ((CD_3_)_2_SO, 295 K): 1.31 s (3H, acetal CH_3_); 1.39 s (3H, acetal CH_3_); 1.84 s (3H, CH_3_); 3.65–3.78 4xd (4H, CH_2_O); 3.81–3.90 m (1H, CH); 5.36 s (1H, CH=); 5.67 s (1H, CH=); 7.66 d (1H, NH) (Figures S2 and S3).

### 2.4 Synthesis of Poly(DHPMA-Acetal) Homopolymer (**A**)

**Scheme 2:**
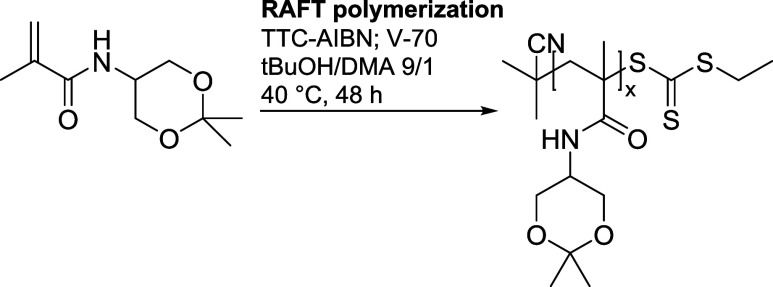
Synthesis of Poly(DHPMA-Acetal) (Homopolymer **A**)

For the RAFT polymerization of DHPMA-acetal (Scheme 2) (160 mg, 0.8 mmol), low-decomposition temperature azo-initiator V-70 (0.83 mg, 2.68 μmol), and TTC-AIBN (1.10 mg, 5.35 μmol) as a chain transfer agent were used in the ratio of monomer/CTA/V-70 = 150/1/0.5. The reaction took place in the mixture of anhydrous DMA and tBuOH (1/9) at a monomer concentration of 0.8 M at 40 °C for 48 h under an argon atmosphere. The resulting poly(DHPMA-acetal) **A** was isolated by precipitation into an excess of dry diethyl ether and purified by precipitation from THF into dry diethyl ether, yielding 83 mg (52%) in the form of a slightly yellow powder.

### 2.5 Synthesis of Poly(DPHMA) Homopolymer (**B**)

The title homopolymer **B** was prepared as described.^33^ Briefly, DHPMA was polymerized via RAFT polymerization in water acidified to pH 1 with HCl using TTC–COOH as a chain transfer agent and VA-044 as an initiator under an argon atmosphere at 40 °C for 24 h.

### 2.6 Synthesis of Diblock Copolymers (**AB1-AB3**)

**Scheme 3:**
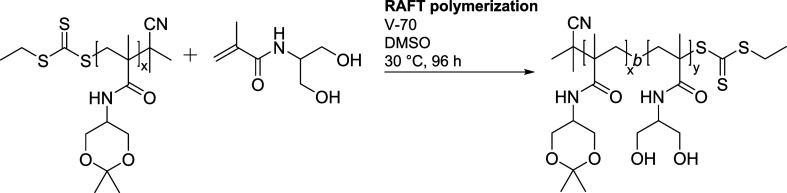
Synthesis of Diblock Copolymers Poly(DHPMA)-*b*-Poly(DHPMA-Acetal) (**AB1– AB3**)

The diblock copolymer **AB1** was prepared via RAFT polymerization of DHPMA by using the homopolymer **A** as a macro-CTA. The TTC-terminated semitelechelic homopolymer **A** (12 mg, 0.78 μmol of TTC groups) was dissolved in DMSO to a concentration of 0.8 M. Then, monomer DHPMA (21 mg; 0.13 mmol) and a low-decomposition temperature azo-initiator V-70 (0.05 mg; 0.16 μmol) were added in the ratio of monomer/macro-CTA/V-70 = 170/1/0.2. The reaction (Scheme 3) took place in an inert atmosphere at 30 °C for 96 h. The resulting diblock copolymer was isolated by precipitation into 20 times the volume excess of diethyl ether and purified by reprecipitation from MeOH to diethyl ether to obtain the diblock copolymer **AB1,** yielding 22 mg (48.0%) in the form of white powder.

Diblock copolymers **AB2** and **AB3** were prepared similarly with different molar ratios of DHPMA/macro-CTA/V-70 (330/1/0.2 for **AB2** and 654/1/0.2 for **AB3**).

### 2.7 Hydrolysis of Acetal Groups

Homopolymer **A** (concentration 2 mg mL^–1^) was incubated in phosphate buffers (0.15 M) at pH 5.0 or 7.4 at 37 °C. The course of acetal groups hydrolysis was determined by ^1^H NMR from the intensity of the signals d and d́ (Figure 8a) at predetermined intervals: 0 min, 40 min, 120 min, 200 min, 320 min, 8 h, 12 h, and 24 h. In parallel, the changes of the transition temperatures (*T*_tr_) were monitored using DLS as described further.

### 2.8 Determination of Hydrodynamic Diameter (*D*_*H*_) and Transition Temperature (*T*_tr_)

The hydrodynamic diameter (*D*_H_) of the polymers was measured by dynamic light scattering (DLS) and static light scattering (SLS) using a Nano-ZS instrument Zetasizer (ZEN3600, Malvern, Malvern, UK) in phosphate buffer (0.1 M, with 0.05 M NaCl; polymer concentration: *c* = 2.0 mg mL^–1^) at 25 °C. The intensity of scattered light was detected at an angle θ = 173°. The wavelength of the laser was λ = 632.8 nm. The values were the mean of at least five independent measurements.

The temperature measurements were performed to investigate the conformation changes of polymer coils in the temperature range 15–70 °C (in 1 °C increments) in PBS (0.15 M, pH 7.4) at a polymer concentration *c* = 2.0 mg mL^–1^ with an equilibration time of 300 s. The DTS(Nano) program was used to evaluate the dynamic light scattering data. The *T*_tr_ characterizing the polymer chain conformational changes (at a specific polymer concentration) resulting in phase separation was estimated from the temperature dependence of the hydrodynamic diameter (*D*_H_) as the onset of the *D*_H_ value increase.

### 2.9 NMR Spectroscopy

Temperature dependences of ^1^H NMR spectra were acquired with a Bruker Avance III 600 spectrometer operating at 600.2 MHz. The width of the 90° pulse was 10 μs, the relaxation delay was 10 s, the acquisition time was 2.18 s, and 16 scans were performed. Each sample was kept for 10 min at the desired temperature before measurement. The integrated intensities were determined with the spectrometer integration software TopSpin. 2D ^1^H–^1^H NOESY NMR spectra were recorded on the same spectrometer with a 4098 Hz spectral window in f_1_ and f_2_ frequency axes, and mixing times in the range of 100–1200 ms. A total of 16 scans was accumulated over 512 t_1_ (evolution time) increments with a relaxation delay of 5 s. The temperature and time dependences of ^1^H spin–spin relaxation times *T*_2_ of HDO and selected proton groups of the copolymer were measured using the CPMG pulse sequence 90°_*x*_-(t_d_-180°_*y*_-t_d_)_*n*_-acquisition. The relaxation delay between scans was 100 s, the acquisition time was 2.84 s with 2 scans. In all measurements, the temperature was maintained constant within ±0.2 °C in the range 12–57 °C (47 °C for the homopolymer) using a BVT 3000 temperature unit. All samples in D_2_O (Euriso-top, 99.9% deuterium) solutions were filled into 5 mm Norell NMR Tubes ST500–7 HT.

### 2.10 Isothermal Titration Calorimetry (ITC)

The solutions of diblock copolymers in PBS were titrated at 37 °C to either pure PBS (5 mg mL^–1^ and 10 mg mL^–1^) or human blood plasma (10 mg mL^–1^), diluted with PBS to 10% (v/v), on a MicroCal ITC 200 instrument (Malvern Panalytical Ltd., UK) in 20 subsequent injections (the first injection of 0.4 μL followed with 19 injections of 2 μL). The measured heat per injection (J) was normalized to the mass of the injected diblock copolymer (g) and plotted vs the concentration of diblock copolymer in the calorimeter cell after the corresponding injection (g L^–1^).

### 2.11 Transmission Electron Microscopy

The morphologies of the nanoparticles at three selected temperatures (5 °C, 37 °C, and 50 °C) were visualized by transmission electron microscopy (TEM). The samples for TEM microscopy were prepared by the fast-drying method described elsewhere.^34−36^ Briefly, the principle of the fast-drying method consists of a very fast removal of solvent from a 2 μL droplet of the nanoparticle suspension deposited on the standard TEM supporting grid. This procedure was applied to nanoparticle suspension kept in the refrigerator (fast drying at 5 °C) and nanoparticle suspension kept in the oven (fast drying at 37 and 50 °C). The dried samples were left to equilibrate at room temperature for 1 h, and then they were observed in a TEM microscope (Tecnai G2 Spirit Twin 12; FEI, Czech Republic) using bright-field imaging at 120 kV.

### 2.12 Differential Scanning Calorimetry (DSC)

DSC analyses were carried out on a DSC Q 2000 (TA Instruments, USA) with nitrogen purge gas (50 cm^3^/min). The instrument was calibrated for temperature and heat flow using indium as a standard. Samples (3–10 mg) of bulk homopolymers were encapsulated in aluminum pans. DSC runs were performed with a ramp rate of 10 °C/min using a heating–cooling–heating cycle from −80 to 150 °C. Two min isothermal plateaus were inserted before and after the cycles. The glass transition temperature (*T*_g_) was defined as a midpoint between the glassy and rubbery branches of the DSC trace.

### 2.13 Cytostatic Effect and Cytotoxicity of the Diblock Copolymers *InVitro*

#### 2.13.1 Cell Lines and Cell Isolation

The mouse T lymphoblastic lymphoma cell line EL4 was purchased from ATCC (Manassas, VA, USA) and cultured in RPMI-1640 medium (Sigma-Aldrich, Czech Republic) with the addition of heat-inactivated fetal bovine serum (10%), 100 U mL^–1^ of penicillin-streptomycin solution, 1 mM sodium pyruvate, 4.5 g L^–1^ of glucose, and 4 mM glutamine. Lewis lung carcinoma LL2 cell line was purchased from ATCC (Manassas, VA, USA) and cultured in Dubeco’s Modified Eagle’s Medium (Sigma-Aldrich, Czech Republic) with the addition of heat-inactivated fetal bovine serum (10%), 100 U mL^–1^ of penicillin-streptomycin solution (Thermo-Fischer, USA), 10 mM HEPES, 4.5 g L^–1^ of glucose, and 2 mM glutamine.

CD8^+^ T cells were isolated from the spleens of female BALB/c mice (8-week-old). Briefly, two BALB/c mice were sacrificed and their spleens were harvested. They were subsequently homogenized using a Gentle-MACS Dissociator (Miltenyi Biotec, Germany) and incubated with ACK-lysis buffer (Thermo Fischer, USA) for 10 min at room temperature. Isolated cells underwent magnetic sorting with negative selection using Naïve CD8^+^ T cell mouse isolation kit (Miltenyi Biotec, Germany).

#### 2.13.2 Cytostatic and Cytotoxic Assays

Tested polymers **AB1**, **AB2,** and **AB3** were diluted in cultivation medium at titrated concentrations and added to the wells with EL4 (5 × 10^3^), LL2 (10^4^), or CD8^+^ T (5 × 10^4^) cells to reach a final incubation volume of 100 μL/well in a 96-well flat-bottom tissue culture plate (Nunc, Denmark). In the case of CD8^+^ T cells, concanavalin A was added to the wells to reach the final concentration of 5 μg mL^–1^. Cells were incubated for 72 h in a humidified 5% CO_2_ atmosphere at 37 °C. Cells incubated in cultivation medium only were used as a negative control; medium only was used as a blank. All experimental conditions were conducted in tetraplicates.

The cytostatic effect was evaluated by [^3^H]-thymidine incorporation assay. Twenty μL of [^3^H]-thymidine (4 μCi mL^–1^; 250× diluted in cultivation medium from stock solution) was added to each well for the last 6 h of incubation. Cells were harvested on a membrane (1450–421 Printed Filtermat, PerkinElmer, USA) by a cell harvester, Harvester 96 (TOMTEC, Germany). Scintillation of the samples was measured on a Microbeta 2450 Microplate counter (PerkinElmer, USA) using plastic melt-on scintillator sheets (Revvity, USA).

The cytotoxic effect was measured by the 3-(4,5-dimethylthiazol-2-yl)-2,5-diphenyltetrazolium bromide (MTT) assay. 120 μL of MTT solution (1 mg mL^–1^) was added to every well after 72 h of incubation with tested samples and aspiration of the supernatant. Cells were incubated with MTT solution for 1 h (5% CO_2_, 37 °C). A 200 μL portion of DMSO was added to each well, and plates were incubated for another 15 min (5% CO_2_, 37 °C). The content of each well was pipetted up and down several times, and absorbance was measured at 530 nm by using an Infinite 200 PRO microplate analyzer (TECAN, Switzerland).

## 3 Results and Discussion

### 3.1 Synthesis of Thermoresponsive Polymers and Copolymers

Diblock copolymers of DHPMA-acetal and DHPMA were synthesized with the intention to prepare thermoresponsive and acidolabile polymerosomes or polymer micelles with a potential application as carriers of anticancer drugs. Three amphiphilic diblock copolymers with constant hydrophobic block length but differing in hydrophilic block lengths (and hence molecular weight) were prepared with the aim to study their associative, thermoresponsive, and pH-sensitive behavior.

#### 3.1.1 Thermoresponsive Block Poly(DHPMA-Acetal)

The synthesis of amphiphilic diblock copolymers intended for micelle or polymerosome formation was performed by RAFT polymerization in two steps. In the first step, the thermoresponsive block poly (DHPMA-acetal) was synthesized. In the second step, the resulting TTC-terminated semitelechelic poly(DHPMA-acetal) homopolymer was used as a macro-CTA for polymerization of the second, fully hydrophilic poly(DHPMA) block. The physicochemical characteristics of the described polymers are listed in Table 1.

**Table 1:** Physicochemical Characterization of the Thermoresponsive Homopolymer and Diblock Copolymers

Sample	*M*_n_^a^	*Đ*^a^	HFOB/HFIL^a^	Conversion (%)^b^	*T*_tr_^c^ (°C)	*D*_H_20 °C (nm)^c^	*D*_H_30 °C (nm)^c^	*D*_H_37 °C (nm)^c^	*D*_H_46 °C (nm)^c^
**A**	13,400	1.04	-	52.2	21–22	4.7	>770	-	-
**B**	21,100	1.05	-	71.1	-	7.6	-	-	-
**AB1**	28,300	1.01	1/0.83	48.0	27–28	8.3	600	120	55
**AB2**	40,300	1.06	1/1.61	51.6	27–29	12.0	700	115	90
**AB3**	63,400	1.33	1/3.10	66.0	26–30	12.9	700	75	60

a Number-average molecular weight, dispersity, and ratio between the hydrophobic (poly(DHPMA-acetal) (HFOB) and hydrophilic block (poly(DHPMA)) (HFIL) were determined by SEC using RI and LS detection.

b Conversion was calculated from the ratio between the measured molecular weight of the given polymer and theoretical molecular weight of the RAFT polymerization.

c Transition temperature and hydrodynamic diameter values were determined using DLS measurement.

The thermoresponsive block had a *M*_w_ = 13,400 g mol^–1^, a dispersity *Đ* = 1.04, and a functionality of TTC groups, i.e., the fraction of polymer chains terminated with TTC functional groups, *f* = 0.95, corresponding to RAFT mechanism of the polymerization. Nevertheless, the conversion of the monomer to polymer was lower than expected, at only 52%. We hypothesize that this is caused probably by the low rate of polymerization of the methacrylamide monomer. Nevertheless, polymerizations performed at higher temperatures or for longer times provided products with a lower TTC end-group functionality.

#### 3.1.2 Amphiphilic Diblock Copolymers

For the synthesis of diblock copolymers, the ratio of CTA/V-70 was increased from 2/1, which was used for the polymerization of DHPMA-acetal, to 5/1 with the intention to minimize the formation of DHPMA homopolymers. The SEC analysis of the diblock copolymers revealed that the copolymers were not contaminated with any significant amount of either the thermoresponsive homopolymer or with the hydrophilic homopolymer (Figure S1). The physicochemical characteristics of the prepared polymers are listed in Table 1.

### 3.2 Solution Behavior of the Thermoresponsive Polymers and Copolymers

#### 3.2.1 Dynamic Light Scattering

**Figure 1:**
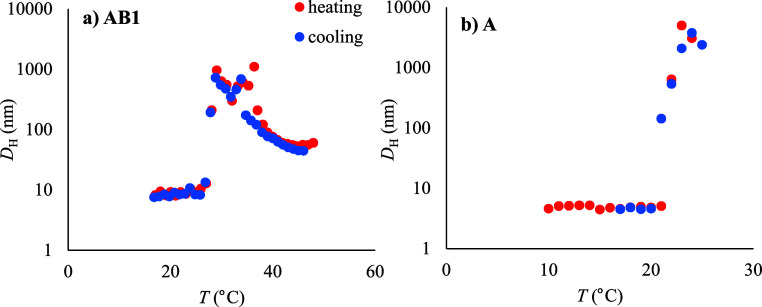
Dependence of the hydrodynamic particle size of a) diblock copolymer (**AB1**) and b) homopolymer **A** on the temperature during heating (red) and cooling (blue).

The thermoresponsive behaviors of both homopolymer **A** and block copolymers **AB1–AB3,** differing in the lengths of the hydrophilic blocks, were studied in detail. Below the transition temperature, *T*_tr_ = 21 °C (at *c* = 2.0 mg mL^–1^), homopolymer **A** occurred in PBS in random coil formation with *D*_H_ = 4.7 nm. Above *T*_tr_, the polymer suddenly precipitated into large macroscopic aggregates with a gradually increasing *D*_H_ > 770 nm, see Table 1 and Figure 1b. The temperature-dependent DLS experiments revealed negligible hysteresis, i.e., the observed *T*_tr_ was the same for the heating and cooling procedures.

The diblock copolymers also exhibited thermoresponsive behavior, but their transition temperatures was about 5–8 °C higher due to the presence of the hydrophilic block. Interestingly, the length of the hydrophilic block had only a negligible effect on *T*_tr_, suggesting almost independent behavior of the thermoresponsive block in the block copolymers, which is in accordance with our previously published results.^37^ Below the transition temperature, all the diblock copolymers were fully soluble, forming polymer coils with *D*_H_ under 10 nm. Immediately above the transition temperature, the copolymers self-assembled into particles with *D*_H_ > 600 nm. Interestingly, while the transition of copolymer **AB1** was sharp, within a 1 °C interval, the copolymers with longer hydrophilic block chains (**AB2** and **AB3)** formed two populations of particles that coexisted just above *T*_tr_ in the temperature intervals of 2 and 4 °C, respectively. Importantly, above this interval, the polymer chains rearranged into supramolecular objects, forming a colloidally stable dispersion that did not sediment even after several hours. We assume that the stability of the dispersion is ensured by the presence of a highly hydrophilic poly(DHPMA) block, which surrounds the hydrophobic core with a stabilizing hydrophilic shell.

Upon further heating of the diblock copolymers, a decrease of particle size was observed. The original large assemblies were gradually reorganized into particles with *D*_H_ below 100 nm (Figures 1a, S4 and S5), whose size did not change over time significantly. Nevertheless, immediately upon fast heating of the sample **AB2** to 45 °C, large particles (>500 nm) were formed, which were reorganized within 2 min into smaller ones with *D*_H_ about 90 nm. However, a small population (less than 5% by volume) of the original large particles coexisted with the smaller particles at this temperature even after 1 h.

We hypothesized that with increasing temperature, the solubility of the hydrophilic block increases, leading to the rearrangement of the whole system. The size of the formed particles was also measured as the system. Practically no hysteresis was observed for the shorter copolymers **AB1** and **AB2** (Figures 1a and S4); only a minimal difference between the cooling and heating experiments was observed for the copolymer **AB3** (Figure S5).

In general, the temperature range of the phase separation is wider for block copolymers **AB2 and AB3** in comparison with homopolymer **A** and diblock copolymer **AB1**. In addition, with the increasing length of the hydrophilic block, this range becomes even wider.

#### 3.2.2 ^1^H NMR Spectra and Fraction *p* of Proton Groups (Units) with Significantly Reduced Mobility

**Figure 2:**
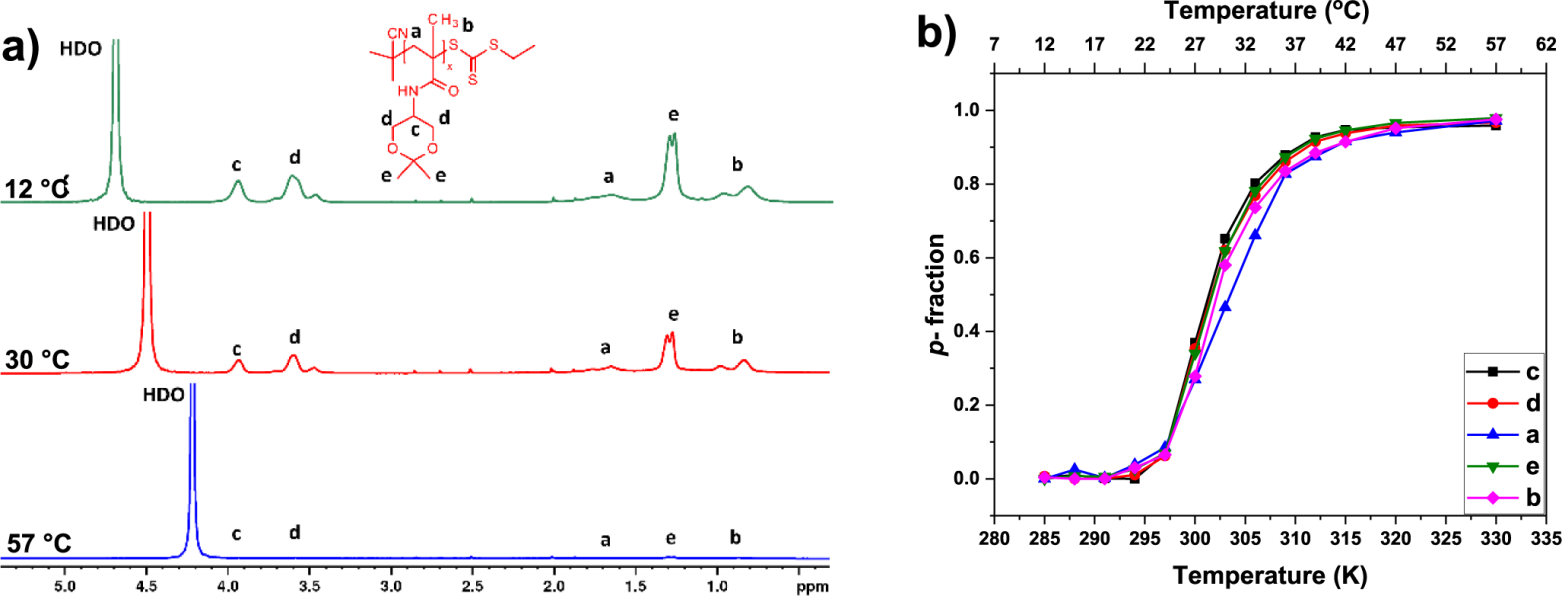
a) ^1^H NMR spectra of homopolymer **A** at 12, 30, and 57 °C. b) Temperature dependences of the fraction *p* as determined for signals of various proton types in D_2_O solution of homopolymer **A** during gradual heating.

^1^H NMR spectroscopy was used to characterize the temperature behavior of the aqueous solutions of homopolymers and copolymers at the molecular level.^17^ Similarly to DLS, temperature dependence of ^1^H NMR spectra was recorded. In Figure 2a, the ^1^H NMR spectra of an aqueous solution of the homopolymer **A,** recorded under the same instrumental conditions at temperatures below *T*_tr_ (12 °C), slightly above *T*_tr_ (30 °C), and significantly above *T*_tr_ (57 °C), are presented together with the signal assignments of various proton types and the chemical structure of homopolymer **A**. The broad signals “a” (δ ≈ 2.00–1.60 ppm) and “b” (δ ≈ 1 ppm) are related to methylene CH_2_ and methyl CH_3_ protons from the homopolymer backbone. Resonance from the homopolymer side chain group C(O)NHC**H**, marked as “c”, was observed at δ ≈ 3.90 ppm. The peak assigned as “d” (δ ≈ 3.60) corresponds to CH_2_ protons, and the signal of the CH_3_ groups “e” is detected at δ = 1.5 ppm. It appears that with increasing temperature, a decrease in intensity and the full disappearance of the polymer signals is observed. This behavior has also been observed in other types of thermoresponsive polymers, such as poly(*N*-isopropylacrylamide) (PNIPAM),^38^ polyoxazolines (POX),^39^ and polymethacrylates containing short oligo(ethylene glycol) (OEG) side chains.^17^ It is obvious that with increasing temperature, the mobility of the polymer segments decreases to such an extent that they escape detection in high-resolution NMR spectra due to the ability to form globular-like structures. For quantitative characterization of changes occurring during the phase transition, the values of the fraction *p* of units with significantly reduced mobility were calculated^17^ using the relation:
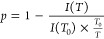
1where *I(T)* is the integrated intensity of the given polymer signal in the spectrum at a given absolute temperature *T* and *I(T*_*0*_*)* is the integrated intensity of this signal when no phase separation of polymer segments occurs. For *T*_*0*_, the temperature was chosen where the integrated intensity of the given signal was the highest, and therefore *p*(*T*_0_) = 0. The results calculated for all homopolymer peaks are summarized in Figure 2b. The temperature dependence of the *p*-fraction of various proton types of homopolymer **A** exhibits practically the same behavior. In all cases, the *p* values start to drastically increase with the temperature from 21 °C to maximum values at 47 °C. This means that the phase transition of the homopolymer starts at 21 °C. All groups of the homopolymer are similarly restricted in their mobility during globule formation. The maximum value of the *p*-fraction (*p*_max_ ≈1) means that 100% of the polymer chains finished the phase transition at around 47 °C. A similar behavior was reported for PNIPAM,^38^ poly(2-(2-methoxyethoxy)ethyl methacrylate)-*co*-*N*-propargylmethacrylamide copolymer,^17^ and POX homopolymer.^40^ Temperature-dependent NMR experiments agree with the results obtained by DLS. The beginning of the changes in the NMR spectra of the homopolymer **A** was detected at 21 °C, similarly as *T*_tr_ that was found with DLS analysis.

**Figure 3:**
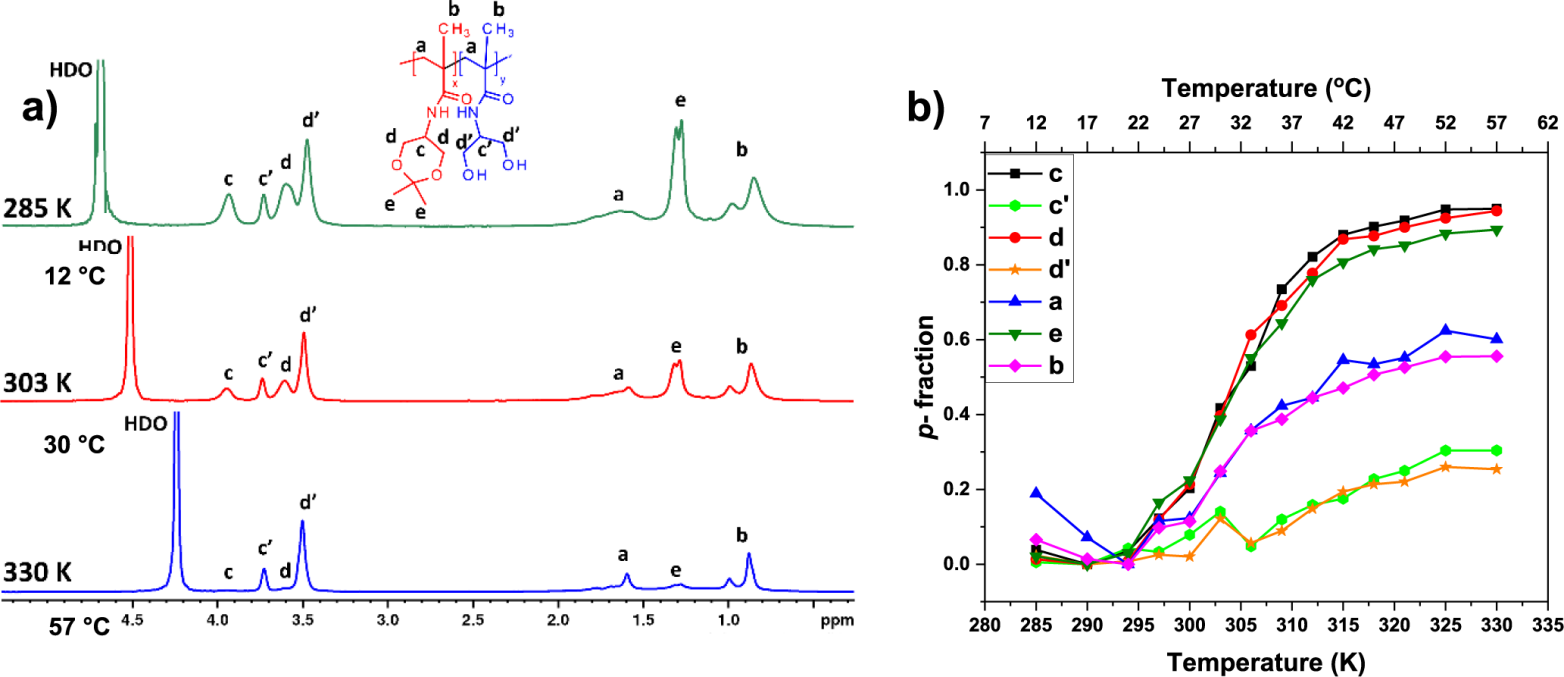
a) ^1^H NMR spectra of copolymer **AB1** at 12, 30, and 57 °C. b) Temperature dependences of the fraction *p* as determined for signals of various proton types in D_2_O solution of copolymer **AB1** during gradual heating.

The same NMR approach was used to study the temperature behavior of **AB1**, **AB2,** and **AB3** copolymer aqueous solutions. High-resolution ^1^H NMR spectra of the D_2_O solution of the block copolymer **AB1,** recorded at three temperatures 12, 30, and 57 °C, under the same instrumental conditions are presented in Figure 3a. Similarly, as in Figure 2a, peak assignments of the various proton types together with the copolymer structure are shown in Figure 3a. The signals of thermoresponsive units (a, b, c, d, and e) are at the same positions as in the spectra of the homopolymer in Figure 2a. Additional two signals of hydrophilic monomer repeating units of the side chain are detected: c’ (CH, δ ≈ 3.75 ppm) and d’ (CH_2_, δ ≈ 3.50 ppm). Peaks from the remaining hydrophilic monomer proton groups (marked as a and b) are overlapped by thermoresponsive backbone signals. Likewise, to **A** homopolymer, the intensity of the peaks of **B** block fully decreases, while the signals of protons of the hydrophilic block remain in the spectrum, even at temperatures above *T*_tr_. This suggests that only block **A** participates in the temperature-induced phase transition. However, the temperature dependencies of the *p*-fraction of various proton types of the **AB1** copolymer (Figure 3b) show that values of *p*-fraction of signals related only to the hydrophilic block (c’, d’) slightly increase with temperature. From the comprehensive point of view, the *p*-values slightly decrease from the starting temperature of measurements to obtain their minimum at 21 °C (which means that polymer segments have highest mobility at this point), next values related only to the groups of thermoresponsive block (c, d, e) increase in a similar way as observed in homopolymer (Figure 2b) to reach the maximum (*p*_max_ ≈ 0.95) at 52 °C. In the case of signals assigned to the hydrophilic block only (c’, d’) *p*-values slightly increase to 52 °C. The *p*_max_≈ 0.20 gives the information that around 20% of the hydrophilic polymer segments participate in phase separation and appear in “solid-like” phase together with the thermoresponsive block. This result is slightly different from that observed previously for the PNIPAM-*b*-PEG^38^ copolymer, where core-to-shell micelles were created above *T*_tr_ and *p*-values for PEG did not exceed *p*_max_≈ 0.09. We hypothesize that the copolymer, above *T*_tr_, creates core-to-shell nanoparticles with a hydrophobic core consisting of the thermoresponsive block and part of the hydrophilic block. Possibly, the hydrophilic block may interact with the thermoresponsive block via intramolecular hydrogen bonds. Similar results were obtained for the **AB2** and **AB3** copolymers.

**Figure 4:**
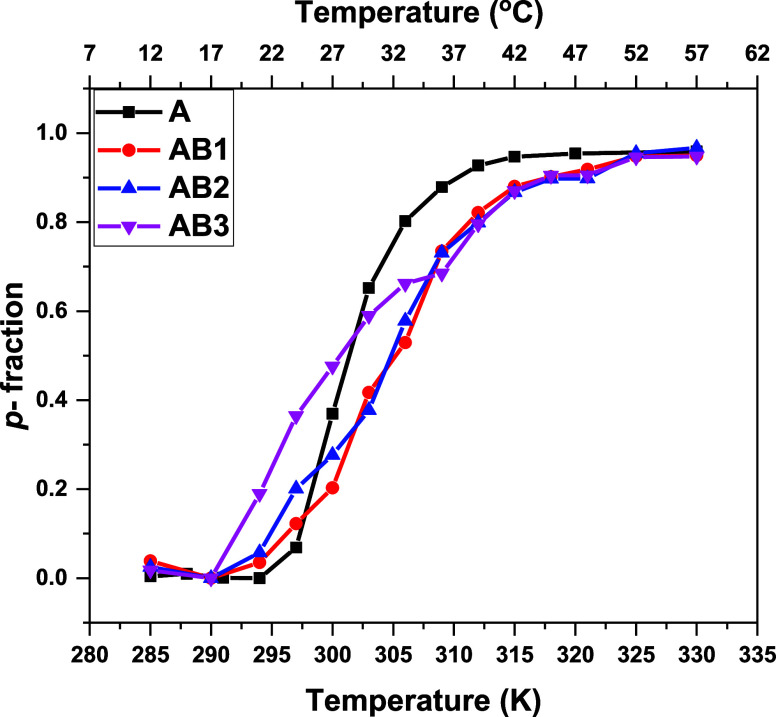
Temperature dependencies of the fraction *p* as determined for signal “**c**” in D_2_O solutions of **A** homopolymer and **AB1**, **AB2**, and **AB3** copolymers during gradual heating.

In order to study the effect of the hydrophilic block on the phase transition of poly(DHPMA-acetal) and due to the fact that the signals related to the thermoresponsive block have in all samples practically the same way of phase transition, signal “**c**” was chosen for further considerations and comparisons. The temperature dependences of the fraction *p* of D_2_O solutions of all investigated polymers are presented in Figure 4. According to the literature, the attachment of a hydrophilic block to the thermoresponsive block should result in an increase of *T*_tr_ with the increasing length of the hydrophilic block,^17,38,41^ This behavior is only partially observed in the case of our block copolymers. Here, the *T*_tr_ (which is estimated as temperature with 0.5*p*_max_ value) of **AB1** and **AB2** increases from 29 °C (for **A**), to 33 °C (**AB1**, **AB2**), but for the copolymer with the longest hydrophilic block, it stays at 29 °C. This rather unexpected behavior of copolymer **AB3** might be possibly caused by a negligible amount of thermoresponsive homopolymer **A** present in the sample. Additionally, the *T*_tr_ values obtained from NMR do not exactly match the *T*_tr_ values obtained from DLS measurements 21 °C for homopolymer **A**, 27 °C for diblock copolymers **AB1** and **AB2,** and 26 °C for diblock copolymer **AB3**. Even if we consider the starting temperature of the phase transition (temperature point at which *p*-values start to increase), which perfectly fits in the case of the homopolymer (21 °C), it fails in copolymers comparisons (17 °C for all copolymers). It can be assumed that the changes in hydration and mobility of the thermoresponsive block observed on a molecular level and reflected by the decrease of the corresponding NMR signals were not sufficient for supramolecular assembly of the individual macromolecules to larger particles, as observed by DLS.

**Figure 5:**
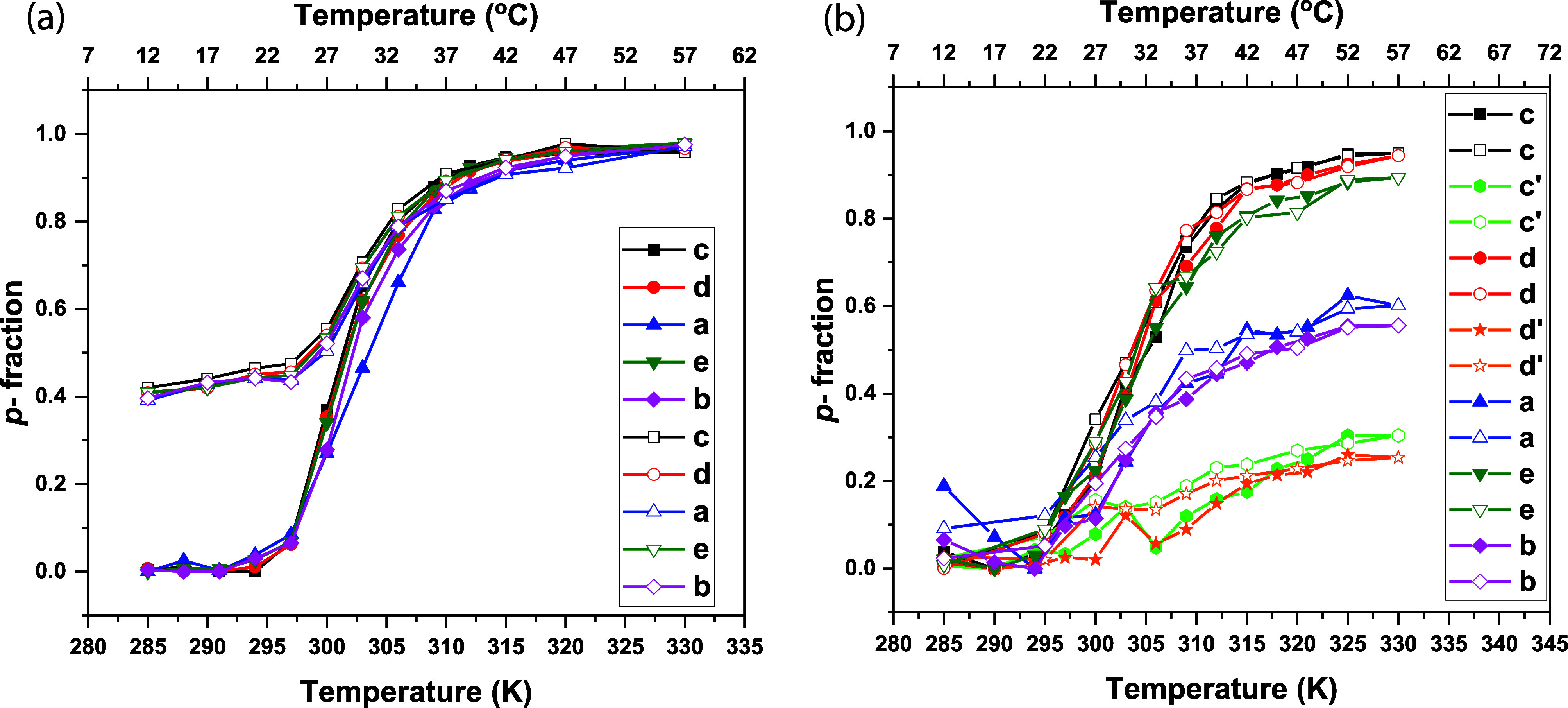
Temperature dependencies of the fraction *p* of the protons with significantly reduced mobility in D_2_O solutions of a) homopolymer **A** and b) diblock copolymer **AB1** during gradual heating (filled symbols) and subsequent gradual cooling (empty symbols).

The reversibility of the phase transition (hysteresis) was verified by measurements of the fraction *p* temperature dependence during gradual cooling performed directly after the heating process. Results obtained for the **A** homopolymer and **AB1** copolymer are shown in Figure 5. Generally, the results obtained in the cooling experiment were the same as those in the heating experiment. The behavior of all proton groups, both in the main polymer chain and in the side chains, is the same in all cases. In the case of the **A** homopolymer (Figure 5a), the *p*-fraction values decrease (from 1 to 0.40) with temperature during gradual cooling. It means that virtually only 60% of the polymer segments reversibly return back to their original state immediately after cooling. This observation seems to reflect the partial irreversibility of the phase transition. A similar effect was already reported for poly(oxazoline) water solutions.^39,40,42^

However, it is necessary to consider that the sample in the NMR cuvette is not stirred. Consequently, a concentration gradient was formed in the sample solution after the heating experiment was finished. This probably led to a lower signal intensity and, hence, virtual irreversibility of the process. Indeed, the signal intensity returned to its original value when the sample was thoroughly mixed.

On the other hand, full reversibility of the phase transition was observed for all three copolymers (the temperature dependence of the *p*-fraction for **AB1** is shown in Figure 5b as an example). Additionally, no hysteresis was observed in the case of the copolymers.

#### 3.2.3 Behavior of Water (HDO) Molecules Determined by ^1^H Spin–Spin Relaxation Times *T*_2_ Study

**Figure 6:**
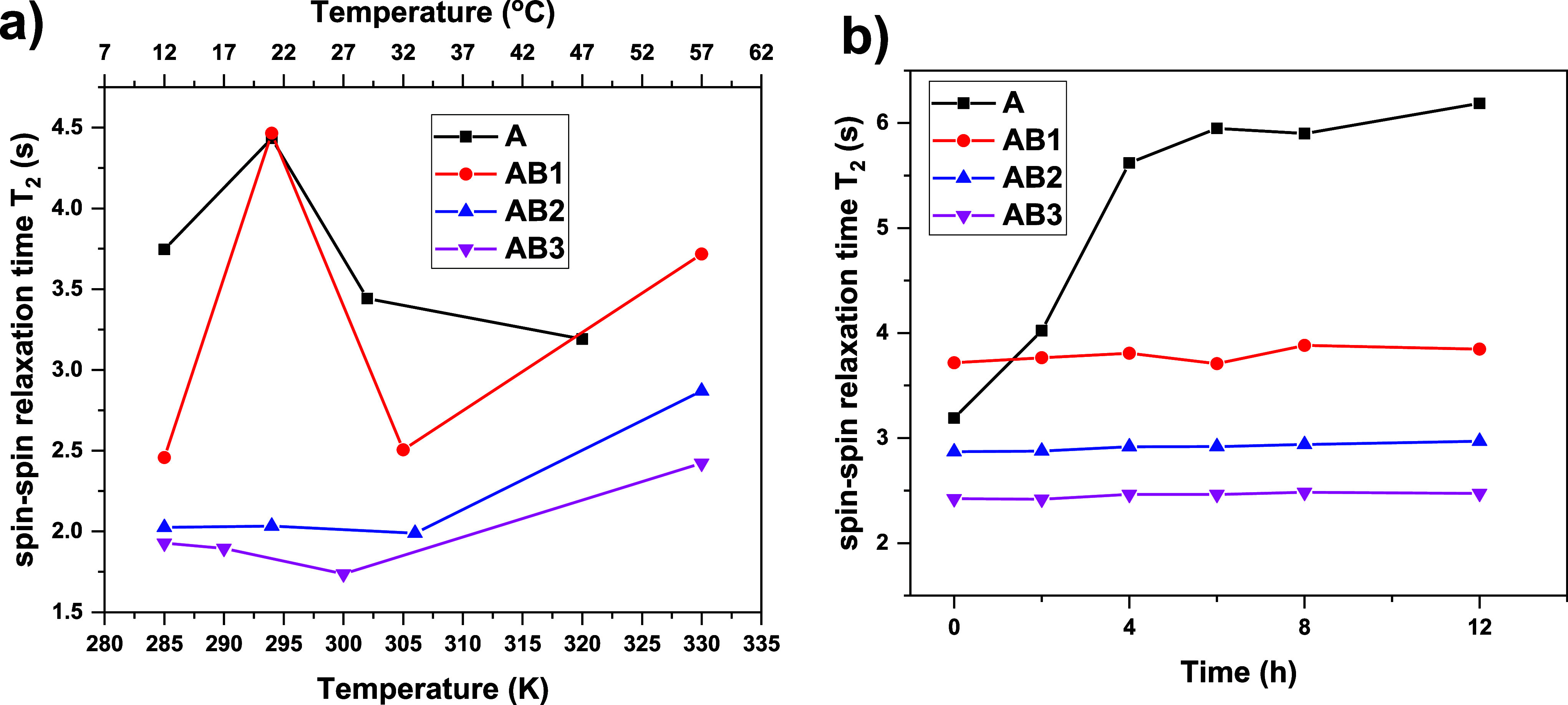
a) Temperature dependence and b) time dependence at 57 °C of ^1^H spin–spin relaxation times *T*_2_ of HDO in D_2_O solutions of the **A**, **AB1**, **AB2,** and **AB3**.

The coil–globule transition process is based on the balance between the polymer–polymer interactions and the polymer–water interactions (especially the changes in hydrogen bonding between water and polymer molecules.^43^ Some information on the behavior of water and polymer–solvent interactions (hydration) during the phase transition in aqueous solutions can be provided by measurements of the mobility of solvent molecules by ^1^H spin–spin relaxation times *T*_*2*_.^17,44^ The temperature and time dependences of *T*_*2*_ relaxation times measured for all polymer solutions are shown in Figure 6a,b, respectively. The experimental temperature points were chosen at temperatures (below *T*_tr_, at the temperature in which *p*-values start to increase, in the middle of the phase transition and above *T*_tr_) based on the temperature dependence of the *p*-fraction (see Figures 2b, 3b and 4). At all temperatures, there was a single line of HDO in the ^1^H NMR spectrum for all investigated samples. Starting *T*_*2*_ values (at 12 °C) decrease with the increasing size of the hydrophilic block (from 3.75 s for the **A** homopolymer to 1.93 s in the **AB3** copolymer solution). It is a logical dependence, which indicates that more water molecules interact with longer hydrophilic polymer chain blocks (more hydrogen bonds between water and polymer molecules). In the pretransition region, different behavior is observed for the **A** and **AB1** samples than for the **AB2** and **AB3** samples. In the case of homopolymer **A** and copolymer **AB1** with a short hydrophilic block, an increase of the *T*_*2*_ values is observed with increasing temperature (directly related to increasing mobility of water molecules) suggesting that A block-water interactions become weaker (releasing more free water molecules); these interactions are replaced by polymer- polymer interactions. Another effect (in this temperature region) is observed for the **AB2** and **AB3** samples, where *T*_*2*_ values remain stable, indicating that for copolymers with longer hydrophilic blocks, the number of hydrogen bonds between the thermoresponsive block and water is much smaller than that between the hydrophilic block and water. The disruption of these hydrogen bonds does not affect the average values of *T*_*2*_. At the third temperature point (in the middle of the transition), a decrease of *T*_*2*_ values is observed in all measured samples. This can be explained by the fact that some water molecules are hidden (or cached) in nanoparticle structures during their formation. For temperatures above *T*_tr_, two different phenomena are observed: first for **A** homopolymer, where a further decrease of the *T*_*2*_ value is observed. It is caused by the formation of aggregates at this temperature (more water molecules are caught). However, the time dependence at the same temperature shows a continuous increase of *T*_2_ values with time. It is connected to the gradual release of water molecules from aggregates in time. A similar effect, as shown in Figure 6b, was observed in PEG-*b*-PNIPAM^38^ and PVCL^45^ water solutions, where the release of originally bound water with time started immediately without any induction period. The *T*_2_ values of the copolymers remain almost unchanged over time, indicating the formation of stable nanoparticles above *T*_tr_ for at least 12 h.

#### 3.2.4 Conformational Changes of Block Copolymer: 2D ^1^H–^1^H NOESY NMR Spectra

**Figure 7:**
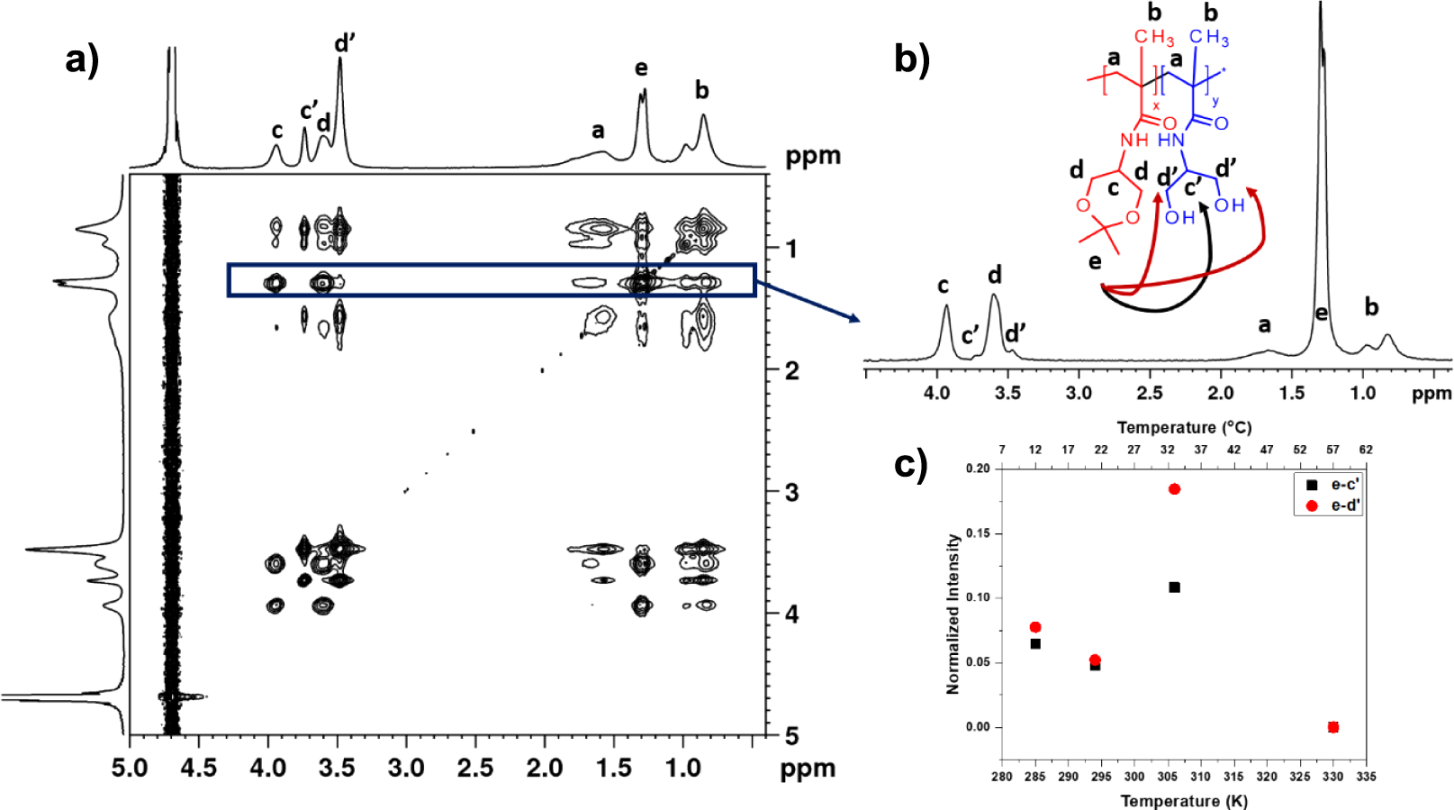
a) 2D NOESY spectrum of **AB2** block copolymer in D2O solution measured at 12 °C with a mixing time of 400 ms. b) 1D slice spectrum extracted from the “e” signal of the NOESY spectrum, together with chemical structure and intermolecular correlations in the **AB2** block copolymer. c) Temperature dependences of integrated intensities of various signals in 1D slices extracted from the signal of protons (“e”) of poly(DHPMA-acetal) units.

2D nuclear Overhauser effect spectroscopy (NOESY) was employed to study the changes occurring during the phase separation and obtain information on the spatial proximity between proton groups of poly(DHPMA-acetal) and poly(DHPMA) units. Generally, NOESY NMR gives the information on the spatial interactions of different nuclear spins in distances to a maximum of 0.5 nm.^38,46^**AB2** sample was chosen and similarly to ^1^H spin–spin relaxation times *T*_*2*_, ^1^H–^1^H NOESY NMR spectra were measured at four temperatures: at 12 °C (starting temperature, below the transition), 21 °C (temperature directly below the transition), 33 °C (slightly above the transition), and 57 °C (significantly above the transition). In the NOESY spectrum measured at 12 °C (Figure 7a), we detected not only cross-peaks between various proton groups within poly (DHPMA-acetal) or poly(DHPMA) units but also weak cross-peaks between side chain “e” protons of P(DHPMA-acetal) units (signal at 1.30 ppm) and poly(DHPMA) side-chain protons (d’, c’). The appearance of all these signals denotes that distances between the respective protons are smaller than 0.5 nm. P(DHPMA-acetal) and poly(DHPMA) units, which are in close proximity, can be both from the same chain of the copolymer and from different copolymer chains. In Figure 7b, the temperature dependences of the integrated intensities of signals in 1D slices, extracted from the signal of “e” protons of P(DHPMA-acetal) of the 2D NOESY spectra measured with the mixing time of 400 ms for a D_2_O solution of the **AB2** diblock copolymer, are shown. From the changes in integral intensity with temperature, it follows that, in the pretransition region, the number of contacts between the two blocks decreases (suggesting the start of chain reorganization already at this temperature). Next, the number of contacts increases in the middle of the transition, even because at this temperature, the *p*-value is 0.5 (this effect is directly related to the formation of nanoparticles). Finally, at temperature above *T*_tr_, no cross-peaks were detected (it was expected due to the full disappearance of signal “e”, *p* = 0.9).

### 3.3 Hydrolysis of the Acetal Groups in the Thermoresponsive Polymers

The isopropylidene ketal groups in the side chains of the thermoresponsive block are known to be susceptible to hydrolysis in acidic aqueous solutions. It was already reported by Huang et al.^16^ that the acid-catalyzed hydrolysis of the ketal groups led to a gradual increase of the transition temperature of the thermoresponsive homopolymer; the complete loss of the thermoresponsive behavior occurred when approximately 30% of the isopropylidene groups were hydrolyzed. Unfortunately, the authors performed the hydrolysis experiments in the pH range 1–4 and did not provide any information about the rate of hydrolysis of the ketal groups at physiologically relevant pH values.

**Figure 8:**
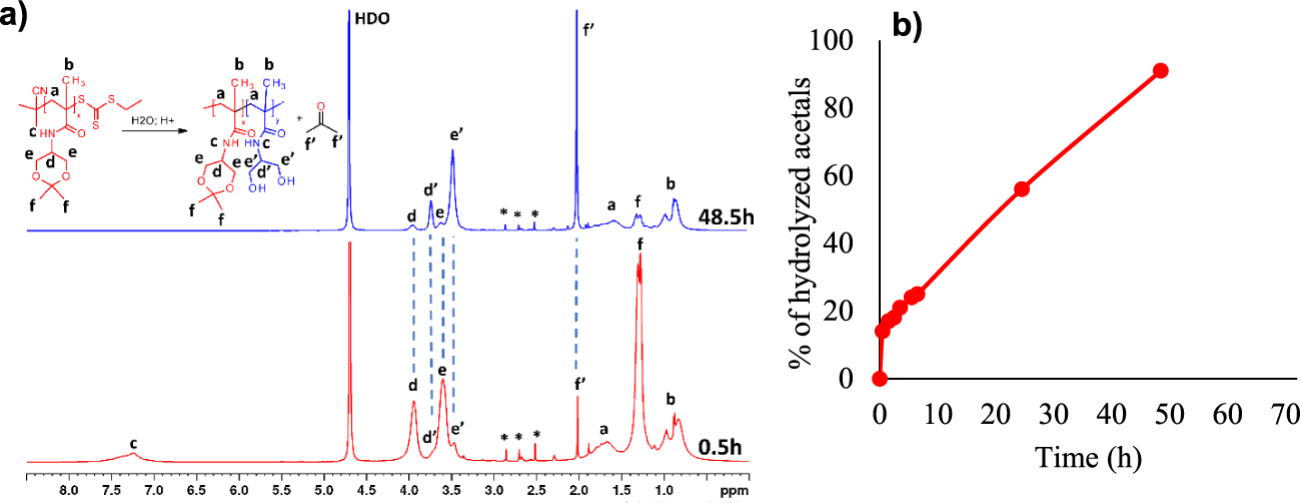
a) ^1^H NMR spectra of homopolymer **A** after 0.5 and 48 h of incubation at pH 5. Signals marked as ″*″ are related to solvent impurities. b) The course of hydrolysis of the ketal groups of homopolymer **A** at pH 5.0.

**Figure 9:**
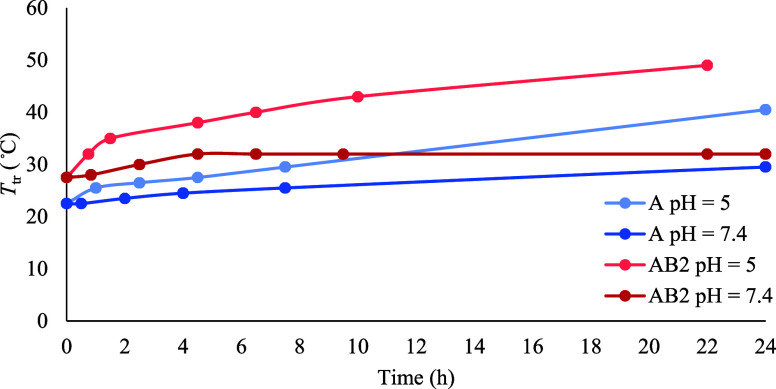
Change of the *T*_tr_ of homopolymer **A** and diblock copolymer **AB2** at pH 5 and 7.4 over time.

In this work, we incubated the homopolymer **A** in aqueous buffers at pH 7.4 corresponding to the pH of the bloodstream and at pH 5.0 mimicking the lysosomal pH. The course of the hydrolysis was monitored using ^1^H NMR spectroscopy (Figure 8a). While there was only a very slow hydrolysis observed at pH 7.4, we found that at pH 5.0, about 55% and 90% of isopropylidene groups were hydrolyzed after 24 and 48 h, respectively (Figure 8b). The removal of the ketal groups resulted in the gradual increase of the transition temperature *T*_tr_ from 21 to 29 °C after 24 h at pH 7.4 and from 21 to 60 °C after 30 h at pH 5.0 (Figure 9).

In the case of the diblock copolymers, hydrolysis of the ketal groups results in the loss of the thermoresponsive behavior and disintegration of the nanoparticles into unimers. This behavior is desirable for nanoparticle drug delivery systems as it ensures final excretion of the polymers from the organism after the corresponding drug cargo is delivered and released at the target site, e.g., a tumor tissue.

Similarly, as in the case of the homopolymer, a gradual increase of the transition temperature from 28 to 49 °C was observed after 24 h of incubation of the diblock copolymer **AB2** in an aqueous buffer at pH 5. Interestingly, at pH 7.4, the transition temperature increased from 28 to 32 °C during the first 5 h; however, it did not grow further in the next 20 h (Figure 9).

We suppose that the initial jump in the *T*_tr_ value might be caused by the hydrolysis of the terminal hydrophobic trithiocarbonate groups originating from the CTA to more hydrophilic thiols.

In general, the susceptibility of the prepared copolymers to pH-dependent hydrolysis will guarantee the elimination of the whole drug delivery system from the organism after the transition temperature increases above 37 °C and the nanoparticles are fully disintegrated.

### 3.4 Critical Aggregation Concentration of the Diblock Copolymers

The dissociation behavior of particles formed by diblock copolymers **AB1**, **AB2,** and **AB3** was studied using ITC at physiological temperature (37 °C). A solution of **AB1–3** in PBS (5 mg mL^–1^) was titrated into pure PBS, and the heat flux from the polymer dilution was measured. The dilution was exothermic for all samples, consistent with hydrophilic polymer solutions in water.^47^

**Figure 10:**
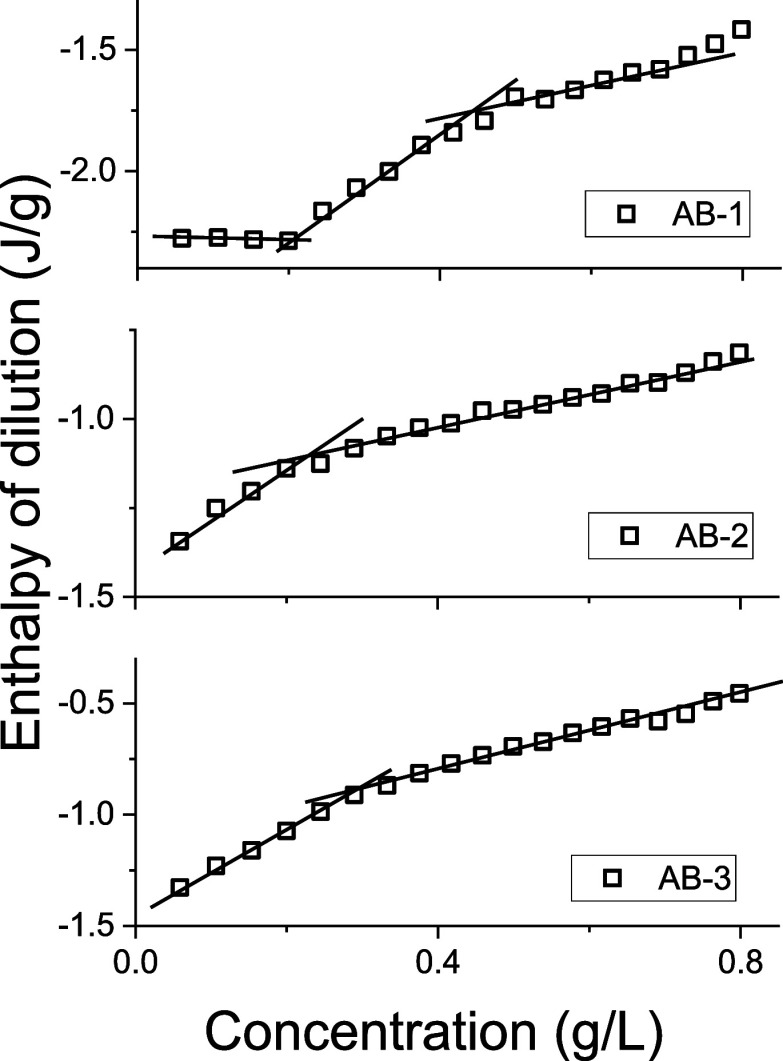
Enthalpy of dilution of 5 mg mL^–1^ diblock copolymers in PBS to pure PBS at 37 °C.

Nonlinear dilution isotherms showed significantly higher enthalpies when diluting from 5 mg mL^–1^ to 0.06–0.4 mg mL^–1^ (Figures 10 and S6), indicating particle dissociation below 0.3 mg mL^–1^. For **AB1**, two inflection points were observed, similar to those of low-molecular weight surfactants:^48^ a positive one at 0.2 mg mL^–1^, likely corresponding to the critical aggregation concentration (CAC), where only dissolved polymers are present, and a negative one at 0.41 mg mL^–1^, marking the dominance of particle populations. Between 0.2 and 0.41 mg mL^–1^, the particles and dissolved polymers coexist in comparable amounts.

For **AB2** and **AB3**, lower overall dilution enthalpies and single negative inflection points were observed at 0.22 mg mL^–1^ and 0.28 mg mL^–1^, respectively. Below these concentrations, particle dissociation became evident. Particle dissolution below 0.4 mg mL^–1^ was confirmed by DLS.

Diblock copolymers (10 mg mL^–1^) were titrated into 10-fold diluted human blood plasma to assess their interaction with blood proteins (Figure S7). Interaction enthalpies plotted on the same *Y*-axis as dilution enthalpies were negligible, indicating that copolymers **AB1–3** exhibit no nonspecific interactions with blood proteins.

### 3.5 Transmission Electron Microscopy

**Figure 11:**
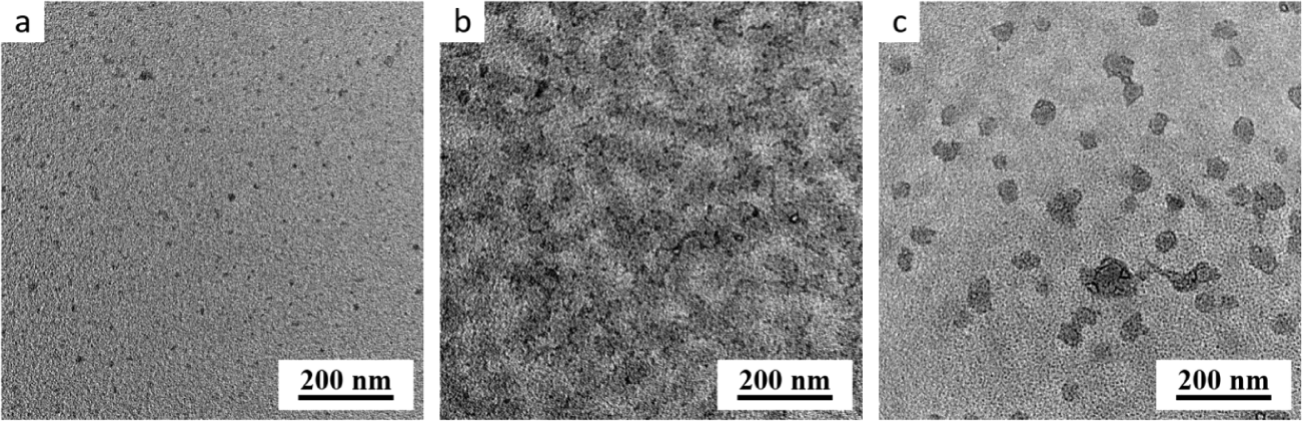
TEM micrographs showing the morphology of the fast-dried particles of **AB1**. The fast drying was performed at (a) 5 °C, (b) 37 °C, and (c) 50 °C.

TEM micrographs (Figure 10) showed the morphology of particles fast-dried at three selected temperatures (5 °C, 37 °C, and 50 °C), which were in good agreement with DLS experiments (cf. Figures 11 and S1–S3). All three types of the studied block copolymers (**AB1**, **AB2**, and **AB3**) exhibited the lowest average size at 5 °C (Figure 11a), the highest size at 37 °C (Figure 11b), and the intermediate size at 50 °C (Figure 11c). Figure 11 shows just the representative TEM micrographs for sample **AB1**, because the results for **AB2** and **AB3** were almost identical, as far as their morphology in the fast-dried state was concerned.

### 3.6 Differential Scanning Calorimetry

It is known that the stability of the micelles depends strongly on the *T*_g_ of the hydrophobic polymer block forming the core of the system.^49^ Therefore, DSC runs were performed on bulk homopolymers **A** and **B**. Both samples are fully amorphous showing one *T*_g_ at 28 °C (Figure S8). Although DSC analysis was performed in bulk, the observed *T*_g_ value is surprisingly equal to the *T*_tr_ value of the copolymers **AB1** and **AB2** found via DLS measurements. Nevertheless, this is most probably just a random coincidence that can be hardly used for interpretation of the associative behavior of the studied copolymers in aqueous solutions.

### 3.7 Cytotoxicity of the Diblock Copolymers In Vitro

**Figure 12:**
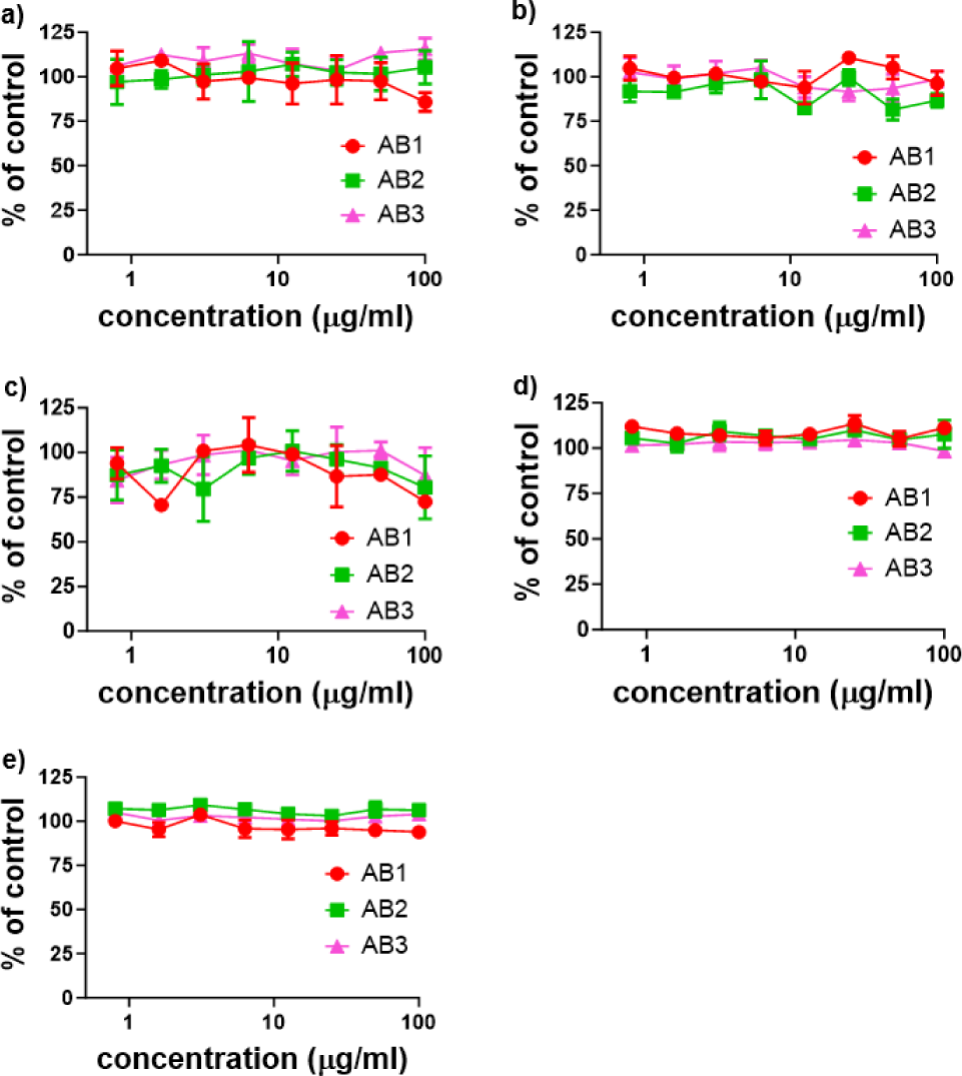
Cytostatic and cytotoxic effects of diblock polymers evaluated in mouse cancer cell lines and purified CD8^+^ T lymphocytes *in vitro*. Cytostatic effect of AB1, AB2, and AB3 determined in a) EL4, b) LL2 cell lines, and c) CD8^+^ T lymphocytes isolated from spleens of BALB/c mice using the [^3^H]-thymidine incorporation assay. Cytotoxic effect of AB1, AB2, and AB3 measured in d) EL4 and e) LL2 cell lines. Each experimental point represents the average value from four wells ± SD. Experiments were performed twice with similar results.

The cytostatic and cytotoxic activities of diblock polymers were evaluated in two mouse cancer cell lines (EL4 lymphoma and LL2 lung carcinoma) as well as in activated mouse CD8^+^ T cells (only cytostatic effect). Results clearly demonstrate that all tested compounds are nontoxic for both cancer cell lines as well as for CD8^+^ T mouse lymphocytes *in vitro,* as any tested polymer compound did not inhibit proliferation or lower the viability of the above-mentioned cells (Figure 12).

## 4 Conclusions

We have successfully synthesized and characterized three thermoresponsive diblock copolymers differing in hydrophobic chain lengths, which self-assemble into nanoparticles in aqueous solutions. These nanoparticles exhibit significant potential as drug delivery systems due to their tunable properties and stimuli-responsive behavior. A comprehensive analysis of their solution behavior and morphology revealed that above ∼28 °C, the diblock copolymers spontaneously form supramolecular assemblies with hydrodynamic diameters exceeding 500 nm. Remarkably, the particle size decreases with increasing temperature, stabilizing at ∼120 nm at 37 °C and ∼60 nm at 46 °C, governed by the hydrophobic-to-hydrophilic block ratio. This thermal transition occurs below physiological temperature (37 °C), making the nanoparticles highly suitable for biomedical applications.

Furthermore, the nanoparticles demonstrate stability under physiological conditions (pH 7.4) and exhibit pH-responsive disintegration at acidic pH 5.0, mimicking lysosomal environments. The hydrolysis of acetal groups within the thermoresponsive block at acidic pH promotes the dissolution of the nanoparticles into fully soluble hydrophilic polymers, enabling the eventual controlled release of the encapsulated cargo. The nanoparticles also remain stable at concentrations above 0.3 mg mL^–1^, ensuring compatibility with physiological conditions. Importantly, we confirmed their noncytotoxicity and minimal interaction with plasma proteins, further supporting their biocompatibility.

The thermoresponsive and pH-sensitive properties of these diblock copolymers facilitate the controlled nanoparticle disassembly and efficient elimination of the polymer from the body. Taken together, these findings underscore the potential of our diblock copolymer-based nanoparticles as versatile platforms for drug delivery, particularly in the antitumor therapy.

## Data Availability

The data supporting this article have been included as part of the Supporting Information.

## Supporting Information Available

SEC chromatograms of polymers, ^1^H NMR spectra of monomers, results from DLS, ITC and DSC analyses (PDF)
