# Supplementary material for: Dual Thermo- and pH-Responsive Polymer Nanoparticle Assemblies for Potential Stimuli-Controlled Drug Delivery
Source: ACS Appl Bio Mater. 2024 Dec 11;8(1):271–84. doi: 10.1021/acsabm.4c01167 (PMC11752510; doi:10.1021/acsabm.4c01167)
Supplement: Supplementary file 1 — mt4c01167_si_001.pdf [file mt4c01167_si_001.pdf]

Supplementary Material

DUAL THERMO- AND pH-RESPONSIVE POLYMER NANOPARTICLE ASSEMBLIES FOR POTENTIAL STIMULI-CONTROLLED DRUG DELIVERY

Sára Pytlíková[a]*, Rafal Konefał[a], Robert Pola[a], Alena Braunová[a], Volodymyr Lobaz[a], Miroslav Šlouf[a], Hynek Beneš[a], Daniil Starenko[b], Kateřina Běhalová[b], Marek Kovář[b], Tomáš Etrych[a], Richard Laga[a], Michal Pechar[a]

[a] *Institute of Macromolecular Chemistry, Czech Academy of Sciences, Heyrovského nám. 2, Prague 6, 162 00, Czech Republic*

[b] *Institute of Microbiology, Czech Academy of Sciences, Vídeňská 1083, Prague 4, 142 00, Czech Republic*

**pytlikova@imc.cas.cz*

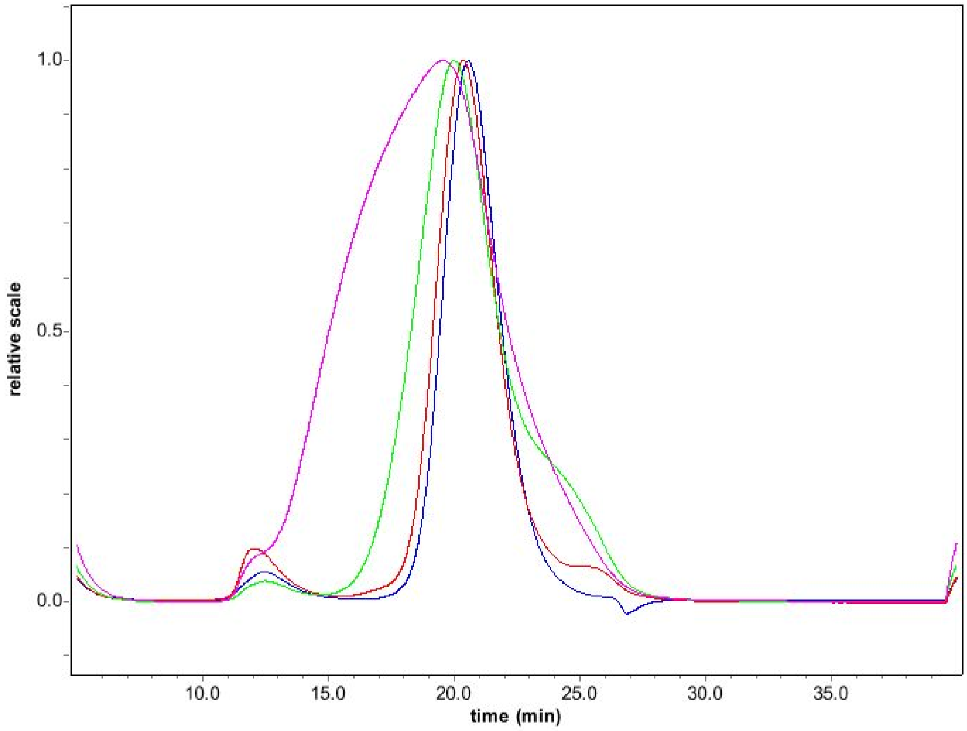

Fig. S1 GPC chromatograms of the thermoresponsive homopolymer poly(DHPMA-acetal) (**A**) (blue) and amphiphilic diblock copolymers varying in the hydrophilic block length poly(DHPMA-acetal)-*b*-poly(DHPMA) (**AB1)** (red), (**AB2)** (green) and (**AB3)** (pink).

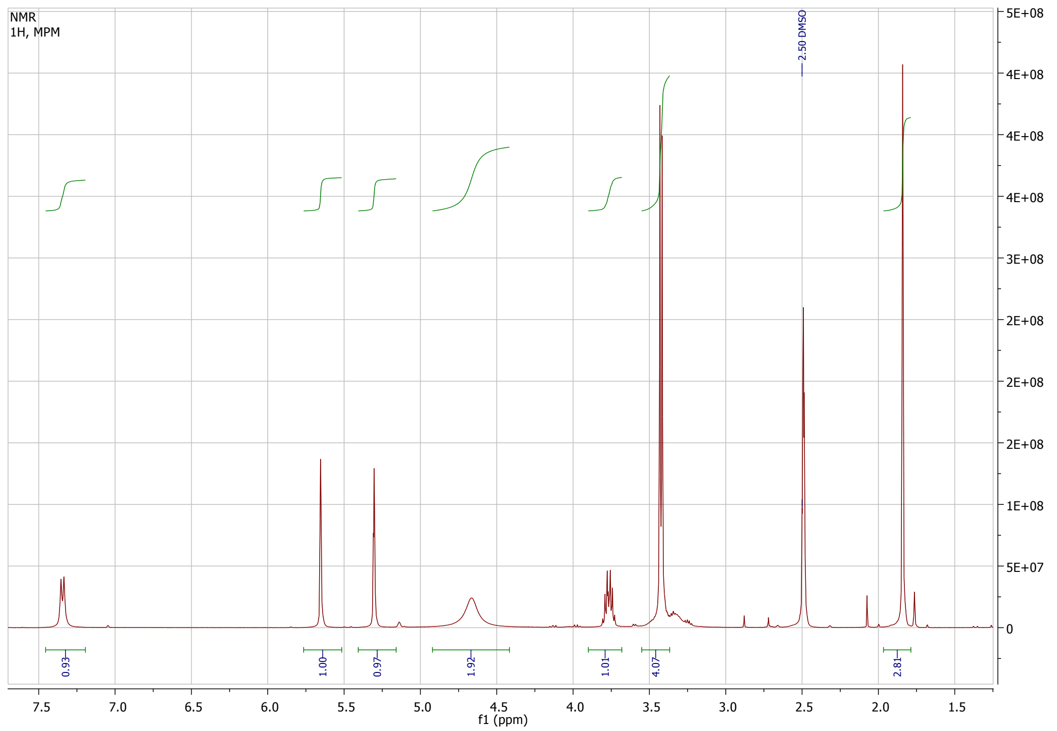

^{1}H NMR (400 MHz, DMSO) δ 7.34 (d, J = 7.9 Hz, 1H, NH), 5.65 (s, 1H, CH2=), 5.41 – 5.16 (m, 1H), 3.77 (dp, J = 7.9, 5.8 Hz, 1H), 3.40 (dd, J = 18.0, 6.4 Hz, 4H), 1.84 (s, 3H).

Fig. S2 ^{1}H NMR spectrum of DHPMA monomer with signal assignments.

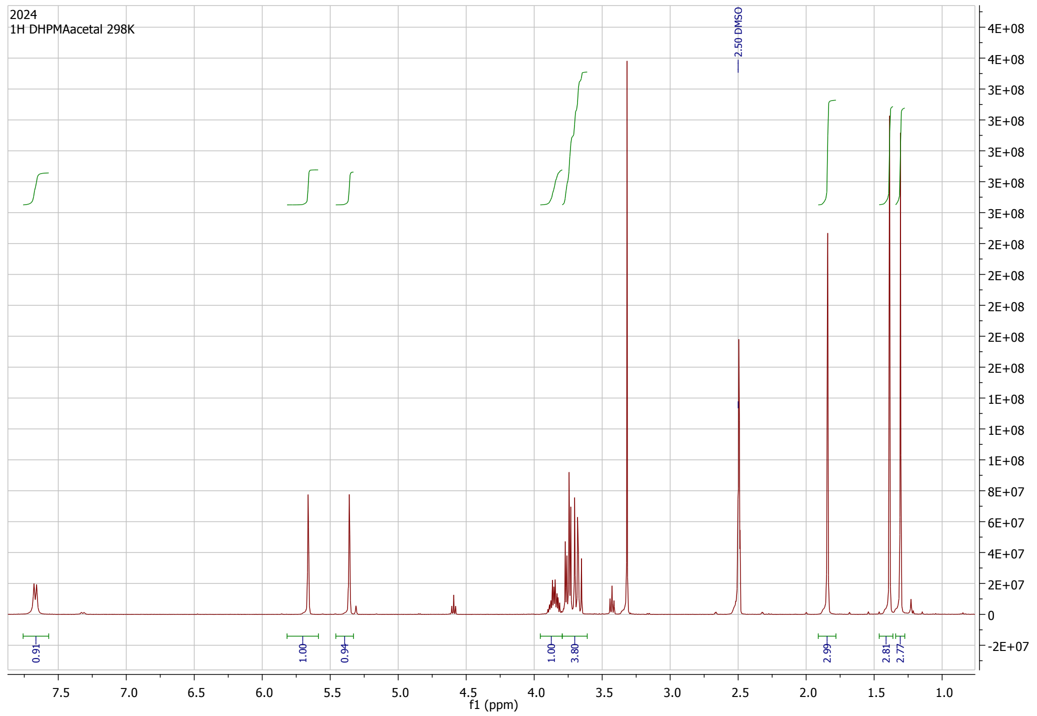

^{1}H NMR (400 MHz, DMSO) δ 7.67 (d, J = 7.8 Hz, 1H, NH), 5.67 (s, 1H, CH=), 5.36 (s, 1H, CH=), 3.96 – 3.79 (m, 1H, CH), 3.79 – 3.61 (m, 4H, CH_2), 1.84 (dd, J = 1.4, 1.0 Hz, 3H, CH_3), 1.39 (s, 3H, CH_3), 1.31 (s, 3H, CH_3).

Fig. S3 ^{1}H NMR spectrum of DHPMA-acetal monomer with signal assignments.

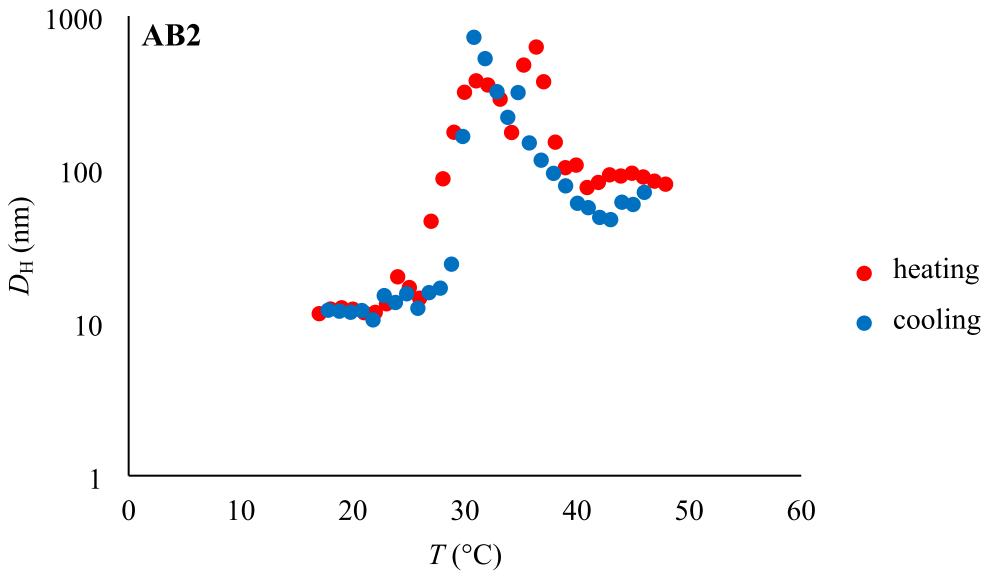

Fig. S4 Dependence of the hydrodynamic particle size of the diblock copolymer (**AB2**) on the temperature during heating (red) and cooling (blue).

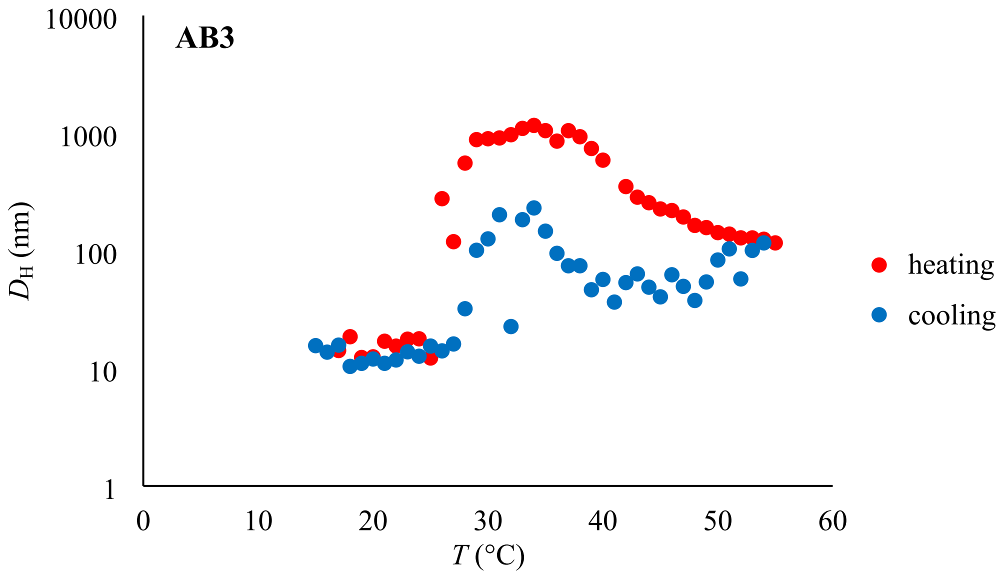

Fig. S5 Dependence of the hydrodynamic particle size of the diblock copolymer (**AB3**) on the temperature during heating (red) and cooling (blue).

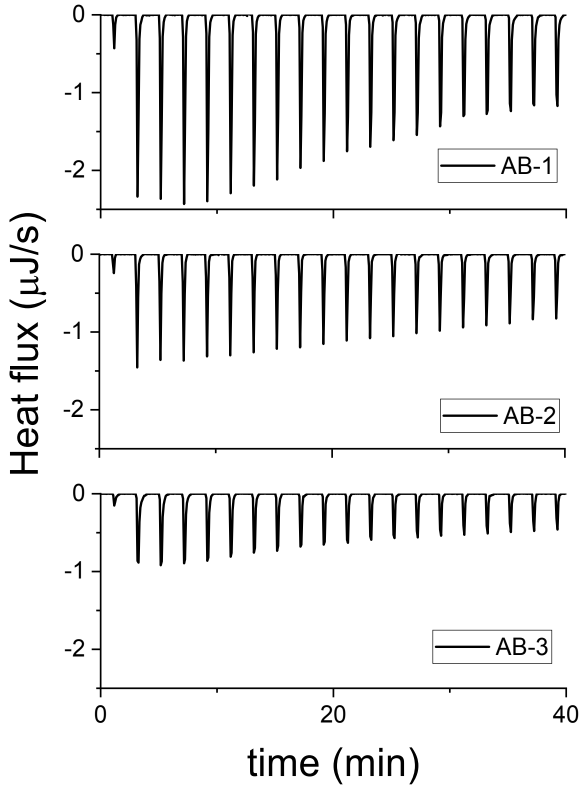

Fig. S6 Raw ITC data (heat flux vs time) for the tritration of 5 mg mL^{-1} diblock copolymers in PBS to pure PBS at 37°C.

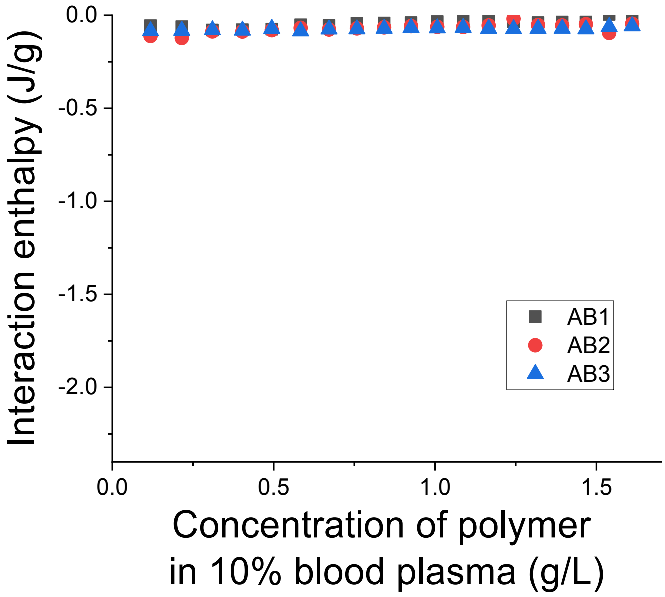

Fig. S7 Enthalpy of the interaction of 10 mg mL^{-1} solution of diblock copolymers with diluted human blood plasma (10% vol/vol) at 37°C.

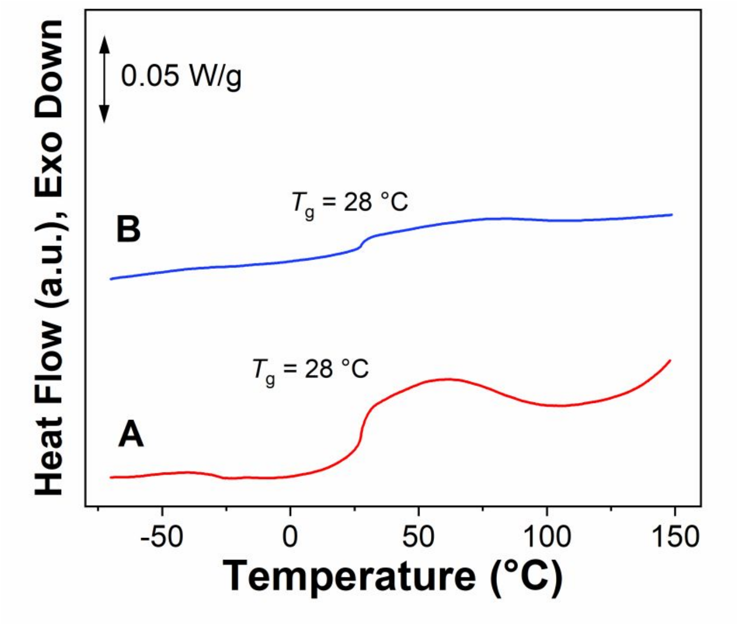

Fig. S8 DSC records (the 2nd heating runs) of bulk homopolymers **A** (red) and **B** (blue).
